# Prediction of Disease-Related Interactions between MicroRNAs and Environmental Factors Based on a Semi-Supervised Classifier

**DOI:** 10.1371/journal.pone.0043425

**Published:** 2012-08-24

**Authors:** Xing Chen, Ming-Xi Liu, Qing-Hua Cui, Gui-Ying Yan

**Affiliations:** 1 National Center for Mathematics and Interdisciplinary Sciences, Chinese Academy of Sciences, Beijing, China; 2 Academy of Mathematics and Systems Science, Chinese Academy of Sciences, Beijing, China; 3 Graduate University of Chinese Academy of Sciences, Beijing, China; 4 Department of Biomedical Informatics, School of Basic Medical Sciences, Peking University, Beijing, China; Uni. of South Florida, United States of America

## Abstract

Accumulated evidence has shown that microRNAs (miRNAs) can functionally interact with a number of environmental factors (EFs) and their interactions critically affect phenotypes and diseases. Therefore, in-silico inference of disease-related miRNA-EF interactions is becoming crucial not only for the understanding of the mechanisms by which miRNAs and EFs contribute to disease, but also for disease diagnosis, treatment, and prognosis. In this paper, we analyzed the human miRNA-EF interaction data and revealed that miRNAs (EFs) with similar functions tend to interact with similar EFs (miRNAs) in the context of a given disease, which suggests a potential way to expand the current relation space of miRNAs, EFs, and diseases. Based on this observation, we further proposed a semi-supervised classifier based method (miREFScan) to predict novel disease-related interactions between miRNAs and EFs. As a result, the leave-one-out cross validation has shown that miREFScan obtained an AUC of 0.9564, indicating that miREFScan has a reliable performance. Moreover, we applied miREFScan to predict acute promyelocytic leukemia-related miRNA-EF interactions. The result shows that forty-nine of the top 1% predictions have been confirmed by experimental literature. In addition, using miREFScan we predicted and publicly released novel miRNA-EF interactions for 97 human diseases. Finally, we believe that miREFScan would be a useful bioinformatic resource for the research about the relationships among miRNAs, EFs, and human diseases.

## Introduction

The complex interactions between genetic factors (GFs) and environmental factors (EFs) contribute jointly to phenotypes and diseases [1]–[4]. The in-silico study of GF-EF interactions have provided great helps in understanding diagnosing, and treating diseases. For example, the research about the interactions between drugs (one class of important EFs) and their targets (GFs) has revealed plenty of important biological insights and promoted drug-target interactions identification [5]–[8]. Moreover, the drug-target interaction prediction methods lay a solid foundation for identifying new indication of approved drugs and hence accelerate new drug development and human medical improvement [9]–[13].

MicroRNAs (miRNAs) are a class of important and newly identified GFs, which regulate the expression of target genes by binding to the 3′ untranslated regions of target mRNAs at the post-transcription level mostly in a negative manner. Increasing studies have shown that miRNAs play critical roles in a number of biological processes, such as cell growth, differentiation, proliferation, development, apoptosis, and metabolism [14]–[16]. Therefore, dysfunction of miRNAs is associated with a wide spectrum of human diseases [17]–[19]. On the other hand, EFs have also been proved to be very important causes to the development of many diseases, especially complex diseases [3], [4], [20]. From above analysis, we can conclude that identifying disease-related miRNA-EF interactions is a very important problem in the computational biology.

In recent years, accumulated studies have shown that miRNAs functionally interact with a number of EFs, such as diet [21], stress [22], cigarette smoke [23], air pollution [24], alcohol [25], drug [26], virus [27], radiation [28] etc, and they work together to affect phenotypes and diseases, including cancer and cardiovascular diseases. For example, hypoxia condition could completely reverse mir-297-mediated repression of VEGFA expression and lead to cancer [29]. Cigarette smoke condensate (CSC) could significantly increase mir-31 expression and activate LOC554202 in normal respiratory epithelia and lung cancer cells, which could result in lung cancer [30]. Besides contributing to the formation of diseases, miRNA-EF interactions could also be used to treat some diseases. For instance, during clinical therapy of advanced stage gastric cancer patients, doxifluridine could significantly impact the expression of mir-181b and mir-21 [31]. Paclitaxel could suppress the expression of mir-29c and contribute to the cure of ovarian cancer [32].

In terms of their importance, therefore, it becomes emergently necessary to analyze and predict miRNA-EF interactions and their relationships with human diseases. In a recent study, we have constructed the miREnvironment database, which contains a comprehensive manually curated collection of experimentally supported interactions among miRNAs, EFs, and phenotypes [33]. Based on the human miRNA-EF interaction data in the miREnvironment database, we previously uncovered a number of biological patterns of miRNA-EF interactions [34]. Moreover, we presented a method to characterize the relationship of EFs based on their interacting miRNAs, a framework to predict the result of cancer treatment by anti-cancer drugs or radiation based on miRNA signatures, and a model to infer novel EF-disease associations based on their interacting miRNAs [34]. These studies present a new dimension of information for miRNA, and suggest a new way for studying the relationships among GFs, EFs, and human diseases [34]. However, this model can not predict ternary relationships among miRNAs, EFs, and diseases together at the same time. To our knowledge, no computational models for potential disease-related miRNA-EF interactions inference have been developed [34]. But such a model is emergently needed. In this study, by analyzing the human miRNA-EF interaction data in the context of a given disease, we revealed that for a given disease, miRNAs with similar functions tend to interact with similar EFs, and vice versa. This finding establishes the theoretical basis for the computational inference of novel disease-related miRNA-EF interactions Based on the above finding, we then developed a semi-supervised classifier based method (miREFScan) to predict new disease-related miRNA-EF interactions. Both leave-one-out cross validation and case study (acute promyelocytic leukemia) have demonstrated that miREFScan has a reliable predictive ability. Finally, we applied miREFScan to predict new miRNA-EF interactions for 97 human diseases, which greatly expanded the relationship space of miRNAs, EFs, and human diseases.

## Materials and Methods

### Materials

Here, we briefly introduce the data and corresponding matrix representation used in this study.

1) The disease-related miRNA-EF interaction matrix *A.*

In order to quantitatively describe known disease-related miRNA-EF interaction, we constructed matrix *A* for each given disease. The entity 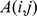 in row *i* column *j* is 1 if the interaction between miRNA *i* and EF *j* contributes to this disease based on the confirmation from miREnvironment database [33], otherwise 0. To construct matrix *A*, we first downloaded the whole dataset from the miREnvironment database (http://cmbi.bjmu.edu.cn/miren, Version of September, 2011), which contains more than 2500 entries in 17 species from 370 publications. Each entry includes information of a miRNA name, an EF name and their related phenotype/disease. This database provides a useful biomedical resource for researchers to study miRNAs, EFs, diseases and the mutual relationship between them [33] and lays the data foundation for disease-related miRNA-EF interactions predictive methods development. We next extracted information of human and obtained 1379 entries to carry on following research. For these 1379 entries, we double-checked the dataset and implemented the following operations: removed the entries with a phenotype named “n/a” and normalized the names of miRNAs, EFs, and human diseases. Finally, we obtained 862 distinct relationships among miRNAs, EFs, and diseases, which include 418 miRNAs, 138 EFs, and 97 diseases. This dataset is regarded as the standard dataset in this study for the performance evaluation of the developed method in both cross validation and case study. This dataset is listed in Table S1. The top four diseases that have largest miRNA-EF interaction dataset are bladder cancer, breast cancer, colon cancer, and xenograft tumor (Figure S1).

2) The EF chemical structure similarity matrix 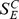 (here, E denotes EF and C denotes chemical structure).

A number of EFs in our dataset are drugs. Chemical structure similarity is often used as an effective drug similarity evaluation measure in drug-related research [9], [35], [36]. Here, we constructed EF chemical structure similarity matrix 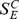 to quantitatively describe the similarity between EFs. The entity 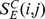 in row *i* column *j* is the chemical structure similarity between EF *i* and *j* if these two EFs are both drugs, otherwise 0. Here, chemical structure similarity is calculated by SIMCOMP [37] based on the information of drug chemical structures from KEGG database [38], PubChem [39], and ChemicalBook (http://www.chemicalbook.com/). Chemical structure similarity score calculated by SIMCOMP is a global ratio between the size of common structures and union structures of two drugs. The chemical structure similarity matrix is shown in Table S2.

3) miRNA functional similarity matrix 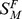 (here, M denotes miRNA and F denotes functional similarity).

We downloaded the miRNA-miRNA functional similarity scores from http://cmbi.bjmu.edu.cn/misim/ [40] in May 2011. Functional similarity between miRNAs was described by matrix 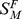. The entity 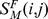 in row *i* column *j* is the functional similarity score between miRNA *i* and *j*, which is calculated based on the observation that miRNAs with similar functions tend to be associated with similar diseases [17].

4) Network-based EF similarity matrix 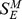 and 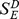 (here, E denotes EF, M (D) denotes similarity based on EF-miRNA (disease) interactions).

**Figure 1:**
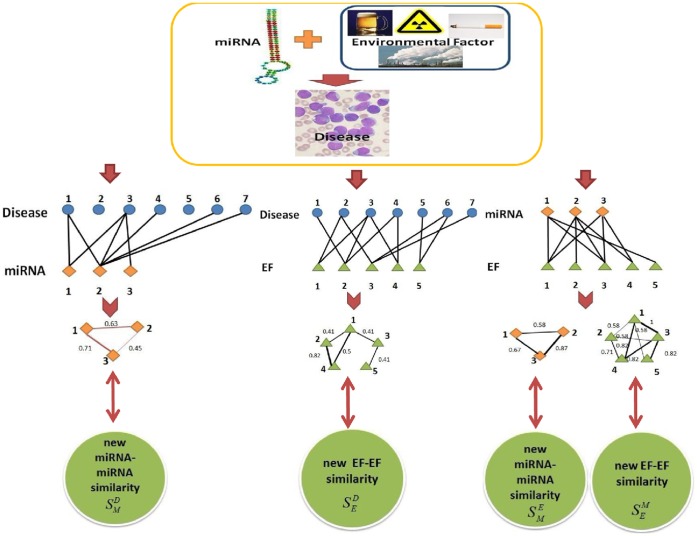
Framework for the calculation of network-based miRNA (EF) similarity.

In order to improve the traditional similarity between EFs, network-based EF similarity matrix 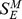 and 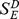 were constructed here. We can obtain disease-miRNA, disease-EF, and EF-miRNA interactions from known disease-related miRNA-EF interactions. Based on these interactions and the underlying assumption that two EFs are more similar if they interact with more common miRNAs (diseases), we can give another similarity measure for EF pairs. The entity 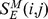 in row *i* column *j* is the number of known miRNAs shared by EF *i* and *j*. Correspondingly, the entity 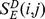 in row *i* column *j* is the number of known disease shared by EF *i* and *j*. The basic idea of new network-based EF similarity has been demonstrated in Figure 1.

5) Network-based miRNA similarity matrix 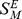 and 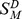 (here, M denotes miRNA, E (D) denotes similarity based on miRNA-EF (disease) interactions).

In order to improve the functional similarity between miRNAs, network-based miRNA similarity matrix 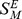 and 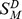 were constructed here in the similar way as the matrix constructed in 4). The entity 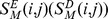 in row *i* column *j* is the number of EFs (diseases) shared by two miRNAs. Figure 1 also demonstrates the basic idea of network-based miRNA similarity.

6) Integrated EF-EF similarity matrix 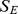 and miRNA-miRNA similarity matrix 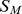 (here, E denotes EF, M denotes miRNA).

In order to accurately describe the similarity between EFs (miRNAs), we constructed the integrated similarity matrix 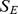 (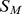) between EF (miRNA) pairs based on traditional drug chemical structure similarity (miRNA functional similarity) and network-based similarity. Here, network-based similarity matrix must be normalized. Take 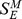 as an example, a diagonal matrix 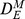 is defined such that 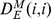 is the sum of row *i* of 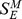 and corresponding normalized matrix is defined as follows: 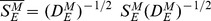, where 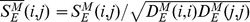. Similar operations are then applied to other three network-based similarity matrix and corresponding normalized matrix 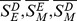 are obtained, respectively. The entity 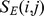 in row *i* and column *j* is integrated similarity between EF i and j, which can be calculated as follows:
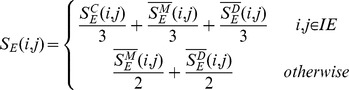
where IE is the set of all the drugs in the EFs and trivial combinatorial coefficients are used here. Similarly, integrated miRNA similarity matrix can also be defined in this manner as follows:



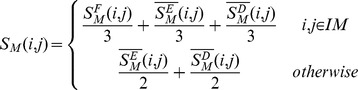



### The Theoretical Basis of miREFScan

The theoretical basis of miREFScan is that miRNA pair interacting with more similar EFs is often more similar, and vice versa. This assumption is referred to as the similar nature of disease-related miRNA-EF interactions in this paper. In order to validate this theoretical basis, we designed and implemented the following two experiments. The similarity used here is the integrated similarity between miRNA (EF) pair that has been defined above.

**Figure 2:**
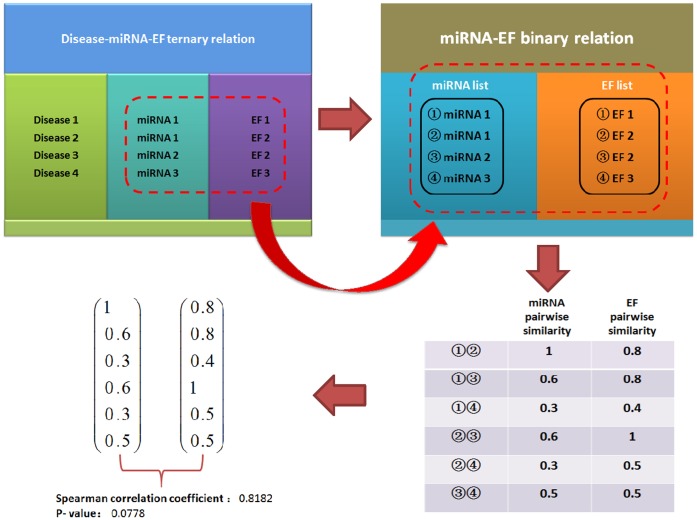
The flow chart of the first experiment for verifying the similarity nature.

For the first experiment, we firstly obtained the binary relation between miRNAs and EFs, which are represented by miRNA and EF list. Then we calculated corresponding pairwise similarity for all the possible miRNA combinations in the miRNAs list. For the EFs list, similar operations were conducted. Thus we obtained two column vectors denoting pairwise similarity for miRNAs and EFs in the same combination order, respectively. At last, we calculated the Spearman correlation coefficient between these two vectors and corresponding p-value. Here, the null hypothesis for calculating p-values is that these two column vectors are not relevant. The above process has been shown in Figure 2. As a result, the Spearman correlation coefficient is 0.2260 and the corresponding p-value is 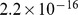, indicating a weak positive correlation between these two vectors. Although the Spearman correlation coefficient is relatively small, the fundamental assumption could be verified considering the fact that the length of vectors is 371, 091.

**Figure 3:**
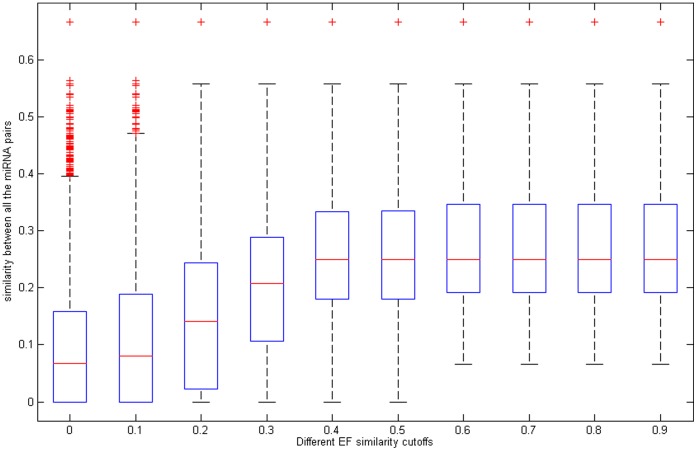
Box plot for the similarity between all the selected miRNA pairs correspond to different EF similarity cutoffs is shown.

Here, we take miRNA as an example for the description simplicity of the second experiment. Firstly, for each miRNA pair, we identified all the EFs interacting with these two miRNAs, separately defined as EF set 1 and EF set 2. Then, the similarity between EF set 1 and 2 (defined as the maximum similarity of all EF pairs combined by any EF in set 1 and 2) was calculated. Thirdly, if the similarity of EF set 1 and 2 was larger than given EF similarity cutoff, we calculated similarity between these two investigated miRNAs. Finally, all the miRNA pairs satisfying the condition that the similarity between corresponding EF sets is larger than a certain cutoff were selected and statistical analysis was implemented. Here the cutoff actually means the degree of the similarity between two EF sets interacting with given miRNAs (i.e. define what is “more similar EFs” in the similarity nature). Box plot for the similarity between all the selected miRNA pairs correspond to different EF similarity cutoffs is shown in Figure 3, which confirms the statement that miRNAs pair interacting with more similar EFs is often more similar. Homologously, the conclusions about EF pairs are also illustrated based on the results in Figure S2.

This basic principle of miREFScan is formulated into two classifiers within the framework of Laplacian Regularized Least Square (LapRLS) in the miRNA and EF space, respectively. Two classifiers are combined to predict potential disease-related miRNA-EF interactions. EF-miRNA pairs with high scores are expected to be associated with disease in interest with high confidence and have priority to be validated in biological experiments.

### Framework of miREFScan

**Figure 4:**
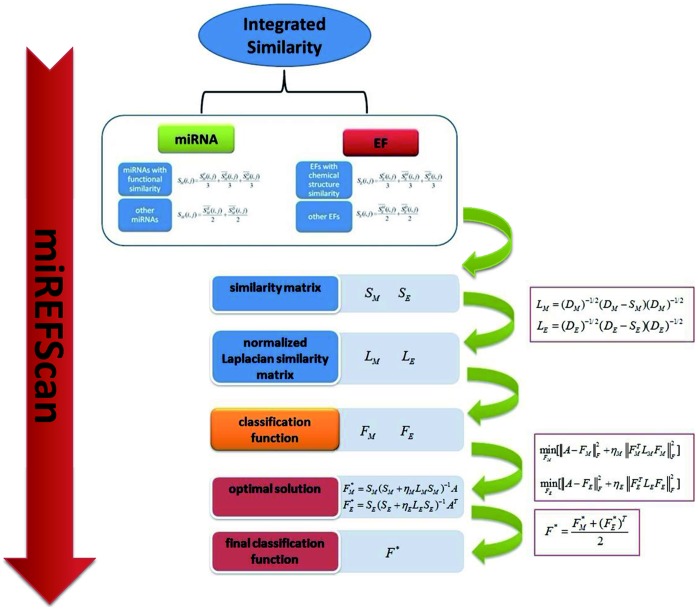
The flowchart of miREFScan includes three steps: calculation of integrated similarity, classifier construction, and classifier combination to obtain final predictive results.

miREFScan aims to select a continuous classification function which could reflect the probability that each miRNA-EF pair is associated with a given disease (Figure 4). It is also expected that the classifiers could meet the following criterions: (1) The classification function complies with the prior miRNA-EF interactions information; (2) This function is smooth over the miRNA and EF space and hence meet similar nature of disease-related miRNA-EF interactions, i.e., when similar miRNA (EF) are combined with the same EF (miRNA), these interactions should obtain similar probability scores. Towards the above purpose, we proposed a method, miREFScan, based on a semi-supervised classifier. miREFScan consists of similarity calculation and employment of the LapRLS, which defines a cost function and minimizes the cost function to obtain classification function. miREFScan works as the following steps. Firstly, traditional and network-based similarity scores are both calculated between EF (miRNA) pairs to quantitatively define the integrated similarity. Secondly, Laplacian operation is applied to the integrated similarity matrix for the employment the framework of LapRLS. Normalized Laplacian similarity matrices are defined as 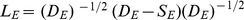 and 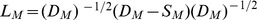, where diagonal matrices 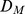 and 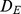 are defined such that 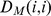 and 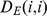 is the sum of the row *i* of 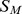 and 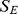, respectively. Then, cost functions are constructed and hence classification functions 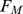 in the miRNA space and 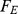 in the EF space are correspondingly obtained by minimizing cost function, respectively. Taking classification function in the miRNA space as an example, optimal classification function can be obtained by solving the following optimization problems: 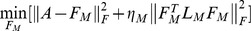, where 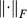 is the Frobenius norm and 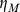 is the trade-off parameter. The solution of the above optimization problem is 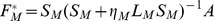 [41], [42]. In the same way, the optimal classifier in the EF space can be obtained as follows: 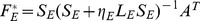. Finally, the classification functions in different spaces are combined together to give the final solution, i.e. 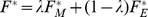, where 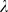 is the weight parameter describing the importance of miRNA and EF space for final prediction results.

## Results

### Leave-one-out Cross Validation

Although three kinds of parameters in miREFScan can be better selected through further cross-validation, here we select the parameter according to some previous studies and will discuss the parameter effect in the next section for simplicity [9], [42]. Therefore, we choose 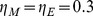 for trade-off parameters in the cost functions [42], 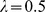 for weight parameter in the final classifier [42] and trivial mean parameter for the similarity integration [9]. We performed cross validation to evaluate the performance of miREFScan. When each cross validation run is implemented, network-based miRNA similarity and EF similarity will be recalculated, i.e. we discard the information from tested disease-related miRNA-EF interactions. miREFScan aims to prioritize candidate miRNA-EF interactions for a specific given disease, i.e. it can not prioritize candidate interaction for all the diseases in the dataset simultaneously. In the gold standard dataset, each disease is associated with 8.89 miRNA-EF interactions on average. This fact means little difference between leave-one-out cross validation and 10-fold cross validation. Many diseases have little known miRNA-EF interactions such as acute myeloid leukemia [43], alcoholic liver disease [44], and lymphoma [45]. Out of 97 diseases investigated in this paper, 32, 17, 12, 9, 3 diseases have 1, 2, 3, 4, 5 known related interactions, respectively. This fact means that we can’t implement multi-fold cross validation for substantial proportion of diseases. Hence, here we use leave-one-out cross validation for performance evaluation.

**Figure 5:**
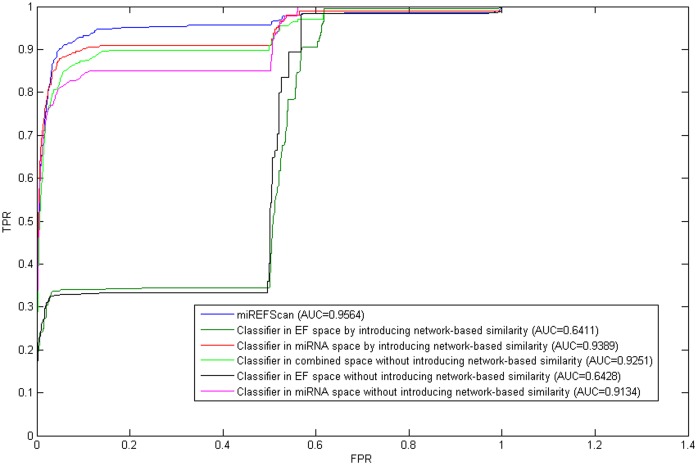
AUC comparison between miREFScan and other methods by leave-one-out cross validation. The result shows that miREFScan has a reliable performance.

When leave-one-out cross validation is implemented, each known miRNA-EF interaction associated with a given disease is taken in turn as test sample and other known interactions associated with the same disease are taken as training samples. Because method developed in this paper is disease-dependent and need known seed interactions, we can’t implement leave-one-out cross validation for the diseases which have only one miRNA-EF relation. For each given disease, candidate sample consists of known left-out interaction and unknown miRNA-EF interactions. Then, we evaluate the method based on the rank of this known left-out interactions in the candidate samples. ROC curve plots true positive rate (sensitivity) versus false positive rate (1-specificity) at different cutoffs. AUC is the area under ROC curve, and AUC = 1 shows perfect performance and 0.5 indicates random performance. Actually, no known disease-related miRNA-EF interactions prediction methods have been reported until now. Hence, we compared miREFScan with some similar methods which either ignore the use of network-based similarity or use the classifier in the single space. An important fact must be pointed out is that these methods with relatively weak predictive actuary are firstly developed in this paper. The aim of comparison between these methods with miREFScan is to demonstrate the reasonability of making full use of network-based similarity and combining classifiers in different spaces into final predictive results. When leave-one-out cross-validation is implemented, ROC curve of each disease can be obtained to assess how well the known miRNA-EF interactions of this disease rank relative to the candidate ones. Therefore, the AUC for each disease is listed in Table S3. Because we will have different AUCs for different diseases, hence we gave a overall AUC for the global evaluation of the methods for disease-related miRNA-EF interactions prediction. The overall AUC comparison with various other methods for all the diseases in gold standard dataset is shown in Figure 5. As a result, miREFScan achieved an AUC of 0.9564 and significantly improved other methods, indicating that miREFScan has a reliable performance.

### The Effect of Parameters on miREFScan Performance

There are three kinds of parameters in miREFScan, including combinatorial coefficients in integrated similarity, trade-off parameters in the cost function and weight parameter in the final classifier.

In Figure 5, the combined classifier without network-based similarity can still obtain a good performance in leave-one-out cross validation (AUC = 0.9251). This observation shows that combinatorial coefficients have little impact on predictive accuracy of miREFScan. In our previous study about drug target interactions prediction, combinatorial parameters were selected in the same way to integrate different drug similarity measures and we also had illustrated the robustness of predictive accuracy to parameter selection [9].

Furthermore, the selection of trade-off parameters is in the same way as the method for drug-target interaction predictions by Xia et al [42], where good predictive performance had been obtained. To further confirm that miREFScan is robust to the selection of trade-off parameters in the cost functions, various values are assigned to 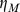 and 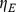 and corresponding AUC of miREFScan is calculated in the framework of leave-one-out cross validation (Table 1). The results show that miREFScan is robust to this parameter.

**Table 1:** AUC in the framework of leave-one-out cross validation schema under different trade-off parameters combination is calculated to confirm that miREFScan is robust to the selection of parameter values.

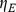 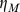	0.001	0.01	0.1	1	10	100	1000
0.001	0.9503	0.9576	0.9544	0.9543	0.9516	0.9573	0.9556
0.01	0.9324	0.9597	0.9578	0.9577	0.9550	0.9606	0.9590
0.1	0.9197	0.9387	0.9565	0.9577	0.9550	0.9606	0.9589
1	0.9137	0.9230	0.9388	0.9573	0.9555	0.9611	0.9595
10	0.9107	0.9171	0.9232	0.9464	0.9510	0.9569	0.9553
100	0.9131	0.9193	0.9243	0.9459	0.9518	0.9581	0.9564
1000	0.9121	0.9183	0.9233	0.9446	0.9507	0.9569	0.9552

For the selection of weight parameter in the final classifier, we admit the fact that there are no good methods to combine different predictive result from corresponding space into the final result. Therefore, we follow the rules used by Xia et al [42], i.e. implement mean operation for results in the different spaces. From Figure 5, we observe that only classifier in miRNA space still obtains a good accuracy, which demonstrates the importance of miRNA dataset. This observation may arise from the fact that most of EFs show little similarity to other EFs. To investigate whether the performance of miREFScan is robust to the selection of this weight parameter, various values from 0.1 to 0.9 are assigned to 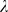 and corresponding AUC is also calculated in the framework of leave-one-out cross validation (Table 2). As a result, predictive accuracy of miREFScan is not sensitive to the selection of weight parameter.

**Table 2:** AUC in the framework of leave-one-out cross validation schema under different weight parameters is calculated to confirm that miREFScan is robust to the selection of parameter values.

*λ*	0.1	0.2	0.3	0.4	0.5	0.6	0.7	0.8	0.9
AUC	0.9347	0.9444	0.9510	0.9547	0.9564	0.9572	0.9575	0.9576	0.9577

### Case Study

Acute promyelocytic leukemia (APL), a subtype of acute myelogenous leukemia, is a cancer of the blood and bone marrow. As a common and highly fatal functional disease, discovering effective therapy ways for APL is definitely an urgent and significant problem in clinical treatment [46]. Although the pathogenesis of APL is very complicated and need to be further comprehended, researchers have confirmed that the combined action of certain miRNAs and EFs is likely to play important roles during the clinical treatment process [47]. For example, 100 nmol/L all-trans retinoic acid could suppress the regulation of several miRNAs, such as let-7a, mir-15a, and mir-16 which is helpful to the therapy of APL [47], and the interaction between mir-21 and arsenic trioxide (ATO), which could regulate ATO-induced cell death, may have a great curative effect for this terrible disease [48]. Based on the above instances, discovering novel miRNA and EF combinations associated with APL is of great importance. Here we predicted novel APL-related miRNA-EF interactions using miREFScan. As a result, for the top 1% candidate samples, 49 novel APL-related miRNA-EF interactions (Table 3) have been confirmed by latest experimental literatures [49]. Especially, mir-16 and let-7a have been proved to be modulated by ATO in the apoptosis process of APL cell NB4 [49]. Consequently, ATO has already been identified as an active agent in the treatment process of APL, both in induction, consolidation, and retrieval therapy stages (http://www.cancer.gov/cancertopics/pdq/treatment/childAML/HealthProfessional/page7). Moreover, these two APL-related miRNA-EF interactions were ranked the 3rd and the 4th in predictive list among more than 50, 000 candidate interactions. Interestingly, the 2nd ranked interaction is between mir-15a and arsenic trioxide, which has been confirmed to produce synergistic apoptosis induced effect in certain primary leukemic cells [50], which means this interaction between miRNA and EF might be a potential therapeutic train of thought for the treatment of many kinds of acute leukemia containing APL. For the 1st ranked prediction between Retinoic Acid and mir-21, we can’t find positive or negative evidences to support or negate this prediction. From known APL-related interactions, we can find three APL-related interactions between Retinoic Acid and let-7a, mir-15a, mir-16, respectively. Considering the fact that these three miRNAs all have relatively high functional similarity with mir-21, we can infer the potential probabilities for 1st ranked interactions. In conclusion, the case study of APL suggests that miREFScan has potential value to discover novel miRNA-EF interactions for given diseases, which will be useful in understanding diseases, diagnosing diseases, and treating diseases.

**Table 3:** Forty-nine APL-related interactions between miRNAs and arsenic trioxide predicted by miREFScan are confirmed by experimental literature [49].

mir-16	let-7a	let-7d	let-7g	mir-181b	mir-155	mir-19a
let-7f	mir-146a	mir-181a	mir-29a	mir-200c	mir-199a	mir-18a
mir-27a	mir-125b	mir-17	mir-126	mir-10a	mir-181c	mir-203
mir-98	mir-143	mir-20b	mir-100	mir-23b	mir-132	mir-1
mir-9	mir-146b	mir-10b	mir-181d	mir-27b	mir-34c	mir-191
mir-125a	mir-372	mir-133b	mir-148a	mir-215	mir-96	mir-149
mir-150	mir-140	mir-214	mir-196a	mir-30c	mir-212	mir-128a

### Predicting Novel Disease–related miRNA-EF Interactions

The leave-one-out cross validation and the case study about acute promyelocytic leukemia have demonstrated that miREFScan has a reliable predictive accuracy. We further applied miREFScan to all the 97 human diseases included in the miREnvironment database. We publicly released the top 100 novel miRNA-EF interactions for each disease for further biological experiment validation (Table S4). These predicted novel relationships among miRNAs, EFs, and human diseases could be useful for biomedical research.

## Discussion

Predicting novel disease-related miRNA-EF interactions is becoming an emergently important problem in bioinformatics, which could not only benefits the understanding of the disease pathogenesis at the miRNA and EF levels, but also plays significant roles in the prognosis, diagnosis, treatment and prevention of disease [34]. In this work, we first observed that miRNAs (EFs) pair interacting with more similar EFs (miRNAs) is often more similar after analyzing the human disease related miRNA-EF interaction data. Based on the above finding, we then developed the miREFScan to predict novel disease-related interactions between miRNAs and EFs based on a semi-supervised classifier in the framework of LapRLS. The result shows that miREFScan has a reliable accuracy of prediction. miREFScan is the first computational tool which can predict ternary relationships among miRNAs, EFs, and diseases together at the same time. It is anticipated that miREFScan would be a useful resource for researches about the relationships among miRNAs, EFs, and human diseases.

The reliable performance of miREFScan could be attributed to the combination of the following several factors. Firstly, a highly reliable set of experimentally supported disease-related miRNA-EF interactions are used as training dataset for prediction. Secondly, from the AUC comparison between miREFScan and classifier in combined space without introducing network-based similarity (Figure 5), we can conclude that proposed integrated similarity between miRNA (EF) pairs improves traditional similarity evaluation measure. More importantly, from the AUC comparison between miREFScan and classifier in single space by introducing network-based similarity (Figure 5), the benefits from combing predictive results in different spaces are significantly shown. Finally, a semi-supervised classifier is constructed to infer novel disease-related miRNA-EF interactions, which could overcome the difficulty of obtaining negative samples in the practical situations. Actually, the advantage of semi-supervised methods over supervised methods has been demonstrated in many previous studies, especially in the practical problems lacking of negative samples. In summary, the reliable performance of miREFScan could be attributed to the fact that miREFScan integrates heterogeneous datasets to capture the relationship between miRNAs, EFs, and diseases.

Of course, miREFScan has some limitations. Firstly, miREFScan can not work for the diseases which do not have known miRNA-EF interactions. In the future, we plan to introduce disease similarity information to solve this problem. Secondly, similarity measures and integration methods from different similarity measures can be further improved. We want to integrate more biological relevant information to define miRNA-miRNA similarity and EF-EF similarity and develop methods such as order statistics used in the ENDEAVOUR [51] and rank fusion algorithm in MCDGPA [52]. Arets et al [51] used order statistics to fuse different prioritizations from multiple heterogeneous datasets into a global ranking for disease gene prioritization to integrate different similarity measures. In MCDGPA for disease gene prioritization, we proposed the rank fusion algorithm to fuse local rank of gene in each module into global rank in the entire network [52]. Thirdly, the performance of miREFScan can be further improved when more disease-related miRNA-EF interaction data are collected in the miREnvironment database. In addition, the final prediction results of miREFScan come from two different classifiers in the spaces of miRNAs and EFs, respectively. How to directly obtain a single classifier or reasonably integrate different classifiers for novel predictions will be an important problem for future research.

## Supporting Information

Figure S1 **The top four largest miRNA-EF interaction networks, which are related with bladder cancer (a), breast cancer (b) colon cancer (c), and Xenograft tumor (d).**
(TIF)

Figure S2 **Box plot for the similarity between all the selected EF pairs correspond to different miRNA similarity cutoffs is shown.**
(TIF)

Table S1 **All the experimentally supported human disease-related miRNA-EF interactions, which is regarded as the gold standard dataset for the performance evaluation in the term of cross validation and case study.**
(XLS)

Table S2 **The chemical structure similarity between all the 138 EFs in the gold standard dataset.**
(XLS)

Table S3 **The AUC for each disease when leave-one-out cross validation is implemented.**
(XLS)

Table S4 **Top 100 novel disease-related miRNA-EF interactions for all the 97 diseases.**
(XLS)
