# Supplementary figures and images for: Prediction of Disease-Related Interactions between MicroRNAs and Environmental Factors Based on a Semi-Supervised Classifier
Source: PLoS One. 2012 Aug 24;7(8):e43425. doi: 10.1371/journal.pone.0043425 (PMC3427386; doi:10.1371/journal.pone.0043425)

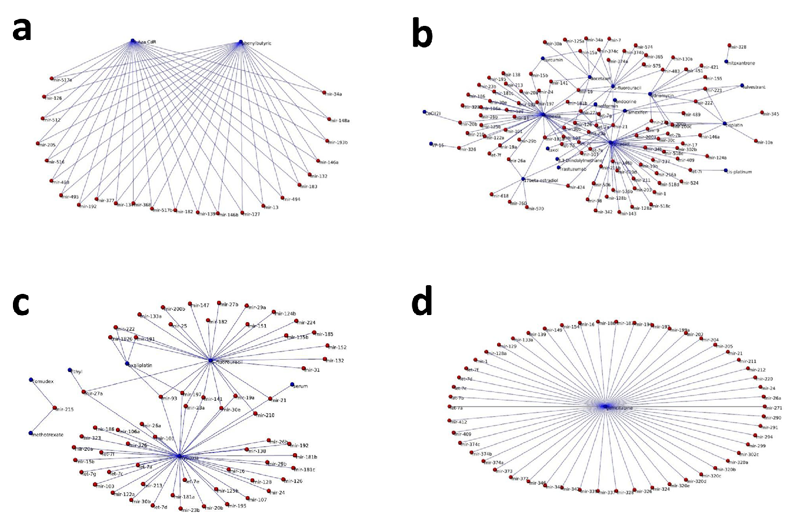

Supplement: Figure S1 — The top four largest miRNA-EF interaction networks, which are related with bladder cancer (a), breast cancer (b) colon cancer (c), and Xenograft tumor (d). (TIF) [file pone.0043425.s001.tif]

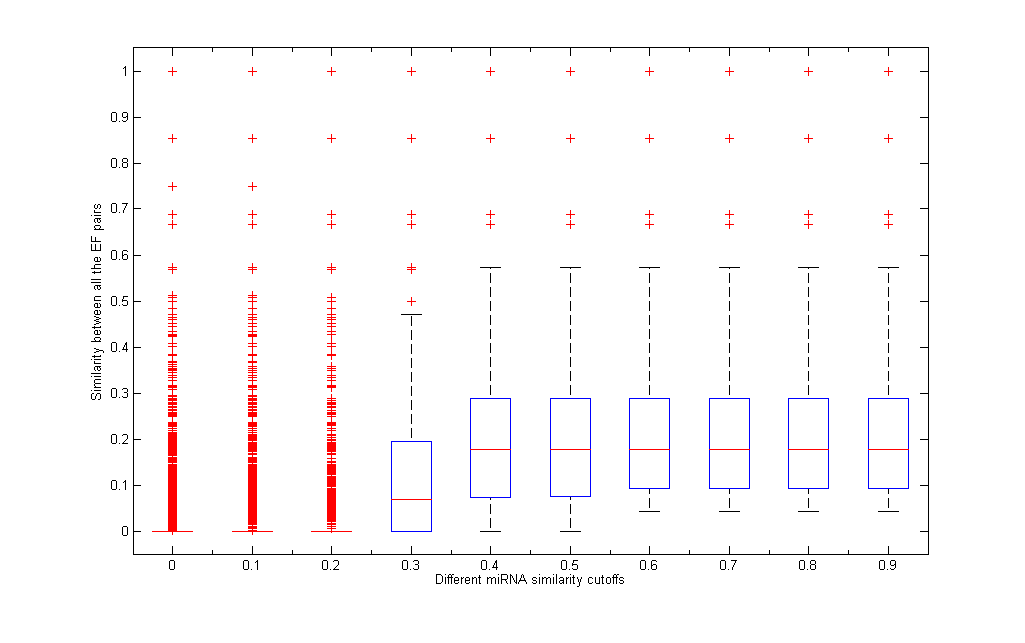

Supplement: Figure S2 — Box plot for the similarity between all the selected EF pairs correspond to different miRNA similarity cutoffs is shown. (TIF) [file pone.0043425.s002.tif]
